# The Effect of Structured Exercise on Short-Term Memory Subsystems: New Insight on Training Activities

**DOI:** 10.3390/ijerph18147545

**Published:** 2021-07-15

**Authors:** Giovanni Ottoboni, Andrea Ceciliani, Alessia Tessari

**Affiliations:** 1 Department of Psychology, University of Bologna, 40127 Bologna, Italy; giovanni.ottoboni@unibo.it; 2 Department for Life Quality, Studies University of Bologna, 47921 Rimini, Italy; andrea.ceciliani@unibo.it

**Keywords:** working memory, sport, exercise, open-skill, closed-skill, children

## Abstract

It has been shown that exercise positively affects cognitive abilities, such as frontal functions and long-term memory processes. We tried to understand whether different exercises (i.e., an open-skill activity, a team game, vs. a closed-skill activity, a circuit) might specifically influence different short-term-memory (STM) subsystems of working memory. We examined the effect of a single bout of open- and closed-skill exercises on three STM tasks (i.e., verbal, visuo-spatial, and motor) in children attending the 3rd and 4th classes at primary school. One group was tested before and after (T0 and T1) an Italian class (control group), one group before and after 30-min exercise on a circuit, and one group before and after 30-min of a team game. The control group presented no improvement. The open-skill activity improved short-term memory performance in all the participants at T1 (*p* < 0.001 for children attending the 3rd class, and *p* = 0.007 for children attending the 4th class). In contrast, closed-skill activity improved short-term memory performance in older children (those attending the 4th class; *p* = 0.046) at T1. Importantly, this finding was found in a school setting and might have ecological validity. Therefore, the exercise protocol here used might help to structure specific training activities for both normal children and those with learning deficits to positively improve short-term memory abilities.

## 1. Introduction

It is well-known that exercise is associated with a reduction in physiological measures of stress, psychological measures of anxiety and depression, and elevations in mood states and psychological well-being [1]. Physical exercise and sport also produce positive effects on arousal, improve attention [2,3,4,5], generally improve cognitive performance [6], with effect size being larger in children than in adults [7], and central high-level cognitive processes (e.g., problem-solving, working memory; [8,9] for a recent review). Memory storage processes were also facilitated by cognitive activation, increasing arousal and allocatable resources induced by specific acute exercise demands (open- vs. closed-skills; [10]). Moreover, moderate and vigorous aerobic exercises promote children’s executive function either after a single bout of exercise [5,6,10,11] or after regular exercise [12,13,14].

Early research investigated the effect of acute exercise on simple cognitive tasks, such as simple and choice reaction time, and visual search in adults ([15], for a review); however, more recent research investigated working memory (WM) as it is more relevant for general cognitive functioning [10,16,17,18]. Recently, research also focused on studying the effects of physical exercise in children [19], highlighting its impact on cognitive functions and executive functions, particularly [20,21]. Physical activity improved cognitive functioning in children aged 3–5 years, especially in the area of working memory [22], and a positive correlation was found between physical activity and better working memory among children aged 8–12 years [23,24]. However, there is little research on acute exercise in children [11]. Moreover, despite the current knowledge of the brain benefits of exercise [25], there are no practical guidelines on optimal practice for children to obtain cognitive improvement.

## 2. This Study

**Figure 1:**
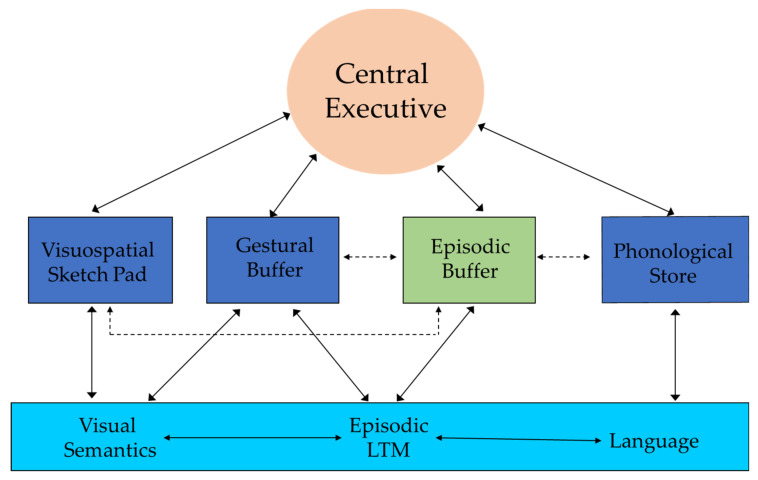
An implemented version of Baddeley’s model [28] of WM is proposed. This includes the two STM subsystems specific for visuo-spatial information and auditory-verbal information postulated by Baddley and an STM subsystem specific for visuo-motor information, i.e., the gestural buffer [29,30,31,32].

In the present study, we investigated the effect of acute exercise (i.e., a single exercise bout) on working memory (hereafter WM) in primary school-aged children as WM plays an essential role in the context of memory and learning and represents a central component in children’s cognition and can explain behavioral difficulties in the educational context [26,27]. Specifically, we investigated exercise effects on the short-term memory (hereafter STM) subsystems, as they represent crucial elements within Baddeley’s WM model ([28] for a review; Figure 1) and have been often left aside in research.

We compared the effects of different types of exercises as only a few studies directly tested such an effect in children ([33] for a recent meta-analysis). Indeed, physical activity can be distributed along a continuum going from open-skill sports (i.e., sports where the environment is constantly changing and movements have to be continually adapted, and where skills are predominantly visuo-perceptual and externally paced, such as basketball, soccer and volleyball and team sport in general) to closed-skill sports (i.e., sports where the performer knows exactly what to do and when, skills are not affected by the environment, and movements follow specific set patterns, such as dance, artistic gym, running, swimming). Open-skill sports require more complex cognitive processes compared to closed-skill ones: the former, for example, need to pick up information rapidly, to exert continuous decision-making, to interconnect different memory structures (e.g., using long-term memory as a tool to extend STM; [34]) and seems to improve cognition more than latter [10] which mainly relies on long-term memory processes such as encoding and retrieval [35] and requires lower cognitive demands and differential neural activation in the brain [36]. They both have a positive effect on cognitive performance in the short- and long-term [37,38,39,40] as they induce release serum brain-derived neurotrophic factor (BDNF; open-skill exercise more than closed-skill exercise [41]). So far, an effect of physical activity on WM capacity has been shown in adults [7], and single bouts of open-skill and closed-skill exercise improved verbal short-term memory in adults [42].

We compared two physically active groups (open- vs. closed-skill exercise) with a cognitively active group and investigated the effects on short-term memory subsystems, specifically, in an ecological context (i.e., during physical education lessons). We hypothesised that an increment in the number of cognitive resources induced by the physical exercise might affect all or part of WM’s subsystems as children might increase storage capacities better using the available resources (following the information processing resources [43]). In particular, we predicted that open-skill activities should generally potentiate short-term memory subsystems at all age stages, as such exercises are characterised by high cognitive demands and require continuous monitoring of the environment. However, given the developmental aspect of WM, we expect that a beneficial effect of closed-skill activities on short-term memory subsystems should emerge, at least in older children.

## 3. Method

### 3.1. Participants

In total, 125 children took part in the experiment (62 children aged 7–8 years and 63 children aged 9–10 years; 55 females) after providing their parents’ written consent. Children belonging to different 3rd and 4th primary school classes were randomly assigned to three experimental groups (control group, open-skill exercise group, closed-skill exercise group), so that the results might not be influenced by factors related to belonging to a specific class. All participants were typically developing children (i.e., children who do not have cognitive delays, specific cognitive impairments, ADHD or specific learning disabilities), and had a normal or corrected-to-normal vision.

### 3.2. Ethical Approval

Ethical approval was granted by the Ethics Committee of the University of Bologna in March 2015, following the Declaration of Helsinki, and parents/tutors provided written informed consent for the children to participate in the study.

### 3.3. Design and Procedure

Participants were administered three short-term memory tasks (a verbal, a spatial and a motor task) before and after a 30-min period filled with three different activities: two experimental groups, where children were required to perform physical activity, and a control sedentary group (i.e., children attended an Italian grammar classroom activity). The experimental conditions consisted of a closed-skill activity (i.e., an agility circuit) and an open-skill activity (i.e., an open-skill ball team game). The exercise intensity (recorded through heart rates with the “Polar” system (Polar H10 heart rate sensor) ranged from 170–180 (±10) bpm. Heartrate monitoring ensured that both exercise conditions were of vigorous aerobic intensity. The agility circuit included a series of exercises (with or without additional tools) performed in a fixed sequence: jumping into four wooden circles placed in a row on the ground, one overturning on a mat, three jumps on both sides of a wooden gym bench, turning around a sport cone and overcoming four obstacles in a row, three jumps on both sides of a bench, one overturning on a mat, slaloming along a course with two sport cones, jumping into wooden circles with changes of direction, rolling on a mat, slaloming along a course with four sport cones, and crawling under an obstacle. The ball team game (named “ball to the king” in Italian) involved two teams, each consisting of three people, which moved in a space corresponding to half a basketball court. The confrontation was two against two, the third child playing the role of the king/queen. She/he had to stay on a bench and receive the ball from her/his teammates. The child who made the point became king/queen, and the roles changed. Children could pass the ball only with their hands and could not run holding the ball in their hand

As for the control group, the test-retest was always proposed in a counterbalanced order before and after a sedentary classroom activity (Italian grammar lesson), on a day when the physical activity lesson was not held.

All activities were performed in a group context (i.e., not individual-based exercise or non-activity).

For the memory tasks, we used an auditory-verbal short-term memory task (i.e., digit span by the WISC-IV—Wechsler Intelligence Scale for Children, IV [44]), a spatial short-term memory task (the spatial Corsi task [45,46]), and a motor short-term memory task ad-hoc developed for this study (we used a sample of simple unilateral meaningless upper limb movements selected among the proximal meaningless gesture of the STIMA—Short Test for Ideo- Motor Apraxia [47]). Regarding the motor task, the experimenter performed all actions using her left hand and children were asked to reproduce them in a mirror-like configuration. A mirror configuration (i.e., right-hand imitation of a left-hand action) was selected because it was demonstrated that there is a natural tendency to use this configuration when imitating [48,49,50].

All the item sequences (i.e., motor, spatial and verbal) were presented for recall in serial order. As soon as the sequence of items was presented, the participants were instructed to reproduce the sequence in the exact order. Participants began with a span length of two items. If they succeeded, they were presented with a sequence of three; if they failed, they repeated another sequence of two. The trial ended when a participant failed to perform one span length twice. The final score (span) for each participant was the number of times on which he/she failed twice, minus one. The participants’ performance was video-recorded. The three memory tasks were presented in a counterbalanced order across participants to minimise order effects.

All participants completed two testing sessions, one before (pre-test) and one immediately after an activity bout (post-test) individually in a quiet space.

### 3.4. Statistical Analysis

Data were treated in a multivariate ANOVA with the between-subjects factors of exercise (open-skill exercise, closed-skill exercise and control activity), age (7–8 years-old vs. 9–10 years-old according to the attended classes, i.e., 3rd class vs. 4th class) and gender (female vs. male) and the within-subjects factors of memory task (auditory-verbal, visuo-spatial and motor span) and time (T0 = pre-exercise vs. T1 = post-exercise). Statistical analysis was conducted using IBM SPSS (version 25; IBM Corp., Armonk, NY, USA). The level of significance was set at *p* ≤ 0.05 for ANOVA. For comparisons, we used a one-tailed *t*-test with Bonferroni correction.

## 4. Results

We ran a preliminary analysis to check whether the groups differed at T0 regarding the three memory tasks, but they did not (all independent samples *t*-test *p* > 0.05).

The ANOVA revealed a significant effect of age (F(1, 112) = 85.13, *p* < 0.001) with the group of 7–8 years-old children showing a lower performance (mean = 4.09, SE = 0.07) than the 9–10 years-old ones (mean = 4.97, SE = 0.07). Children also showed an effect of time (F(1, 112) = 19.59, *p* < 0.001), as they performed overall better at T1, the post-exercise (T0: mean = 4.41, SE = 0.05 vs. T1: mean = 4.65, SE = 0.06), and of memory task (F(1.96, 112) = 185.63, *p* < 0.001), as they performed better in the verbal (mean = 5.28, SE = 0.088) and visuo-spatial tasks (mean = 5.02, SE = 0.079) than in the motor one (mean = 3.29, SE = 0.072). Gender and exercise were not significant (*p* > 0.05).

**Figure 2:**
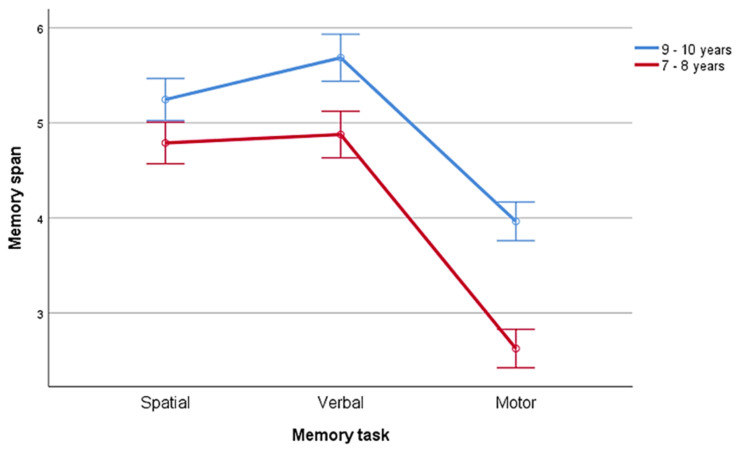
Memory spans are reported according to the three memory tasks and ages. Bars represent standard errors.

Memory task × age was significant (F(1.96,112) = 7.81, *p* = 0.001) as the group aged 9–10 performed better in the verbal task (mean = 5.69, SE = 0.125) compared to both the spatial mean = 5.25, SE = 0.112, *t*(62) = 2.82, *p* = 0.003) and motor (mean = 53.96, SD = 0.103, T(62) = 10.08, *p* < 0.001) tasks, and in the spatial compared to the motors one (*t*(62) = 9.38, *p* < 0.001). On the contrary, the group aged 7–8 did not show differences between the spatial (mean = 4.79, SE = 0.111) and the verbal tasks (mean = 4.88, SE = 0.124): (*t*(60) = 0.884 *p* > 0.05); however both the verbal (*t*(60) = 16.32, *p* < 0.001) and the spatial task (*t*(60) = 14.74, *p* < 0.001) resulted in a better performance compared to the motor one (mean = 2.65, SE = 0.102). See Figure 2.

**Figure 3:**
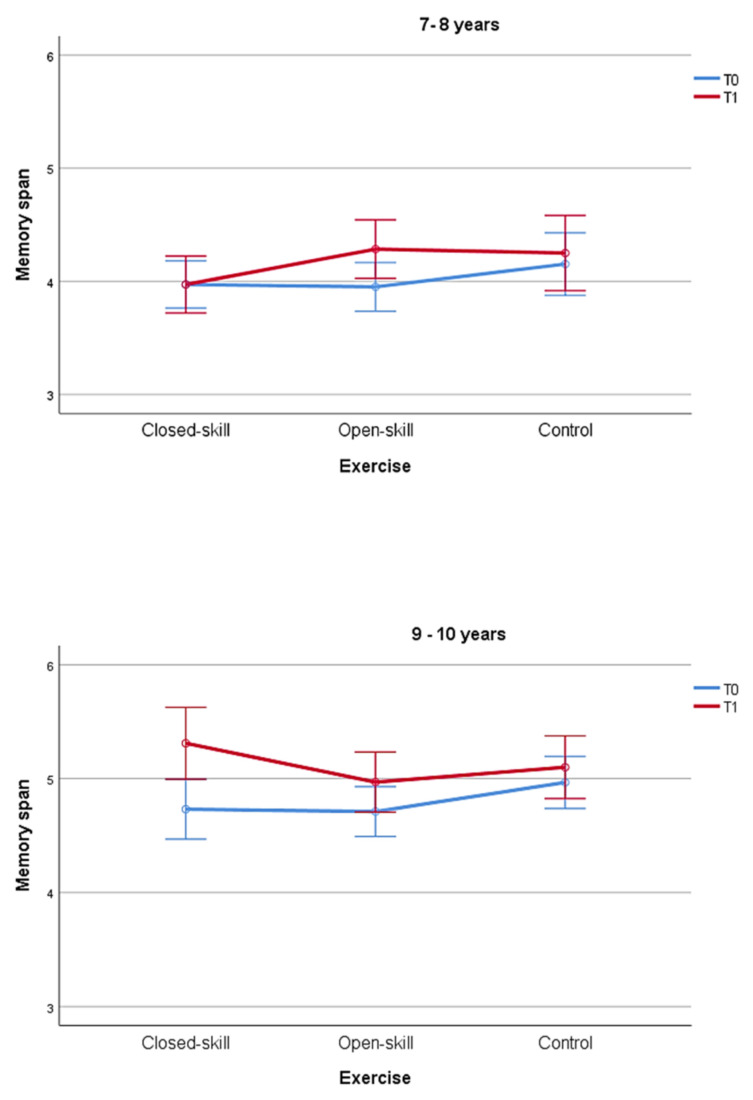
Memory spans are reported according to the three types of exercise, ages, and time. Bars represent standard errors.

Most importantly, the time × age × exercise interaction was significant (F(2, 112) = 3.79, *p* = 0.026): in the group aged 7–8 years, we found a positive effect of the open-skill exercise with a better performance at T1, post-exercise retest (one-tailed corrected *t*-test, *t*(22) = 3.86, *p* < 0.001), but no effect of both closed-skill and control activities (*p* > 0.05). On the contrary, in older children (aged 9–10 years) we found a positive effect of both open-skill activity (one-tailed corrected *t*-test, *t*(20) = 2.68, *p =* 0.007) and closed-skill activity (one-tailed *t*-test, *t*(21) = 1.76, *p* = 0.046) on the cognitive performance at T1, post-exercise retest, but no improvement in the control condition (*p* > 0.05). See Figure 3.

## 5. Discussion

We investigated the effect of one bout of open- or closed-skill exercise on STM subsystems in primary school-aged children. We concentrated on STM subsystems due to the role they play in WM, which is crucial in supporting cognition.

The main result is related to the beneficial effect of one bout of aerobic exercise on memory performance in children. Open-skill exercise was beneficial in both age groups; on the contrary, closed-skill exercise starts to be effective for older children (9–10 years). Significantly, STM performance improved only in the two groups performing physical exercise. On the contrary, the control condition did not improve at both ages, suggesting that the benefit for memory performance found in the two physically active groups cannot be confounded with a learning effect.

We also found an effect of memory task × age: The group aged 7–8 performed better in the verbal and the visuo-spatial STM compared to motor one. However, in the older group (aged 9–10 years), a gradient emerged among the verbal STM (the best), the visuospatial (in the middle) and the motor (one). This is in line with the different developmental trajectories of the WM components. There is little evidence of a global relationship between the motor and cognitive domain in typically developing children aged 4–16 years [51] and the correlation among developmental domains (such as motor, cognition, and language) is not strong. Stable and associated developmental pathways for language and motor performance has been found from 3–5 years of age, but the relationship changes from early to later preschool years [52]. Moreover, even though the basic modular structure of WM is present from 6 years of age, a multi-component model of WM [53] showed that each WM component has a unique developmental trajectory [54].

Regarding the main results, in accordance with the cognitive engagement hypothesis [33], open-skill exercise was expected to produce the most cognitive benefits on WM. Open-skill exercise results are in line with literature often reporting a beneficial effect of intermediate intensity exercise on many WM tasks [16]. However, closed-skill exercises, requiring less cognitive engagement and are already known to be less effective, gave interesting results compared to literature [10]. Indeed, this study shows for the first time that closed-skill activity may also have a positive effect on prefrontal ability, such as STM abilities.

Our results are in line with a previous pilot study in children aged 6–8 years [55], where one bout of open-skill exercise produced verbal memory benefits, but they also differ as we did not find an improvement on motor STM for the closed-skill exercise. However, the two studies present differences. On the one hand, participants’ age differs: Participants are older in this study and might have passed the psychomotor developmental phase in which motor skills are more relevant than complex verbal and visuospatial ones [52]: motor abilities might have become stable, and exercise is not effective according to the age dependence hypothesis [25,56] (see below for more details). On the other hand, the numerosity of the sample differs: this study tested a higher number of participants, and the effect in O’Brien and colleagues’ study [55] might have been spurious. Thus, this study is essential as, for the first time, it shows a positive effect of acute bouts of exercise on STM in a large sample of participants (compared to previous literature that did not [57,58]).

Indeed, a positive effect of bouts of aerobic [16] and anaerobic exercise [59] have been found to improve long-term memory performance. For example, Pesce and colleagues [10] assessed the effect of different types of aerobic exercise on recall in pre-adolescents (aged 11–12 years). They found improved performance on a delayed recall task (requiring a greater involvement of WM) after both a 40-min session of closed-skill exercise or open-skill exercise but only an effect of open-skill activity for immediate recall.

Compared to other studies that did not display any effect on WM speed processes ([16] for a meta-analysis), this study shows that bouts of intermediate intensity exercise influence other aspects of WM, i.e., the STM subsystems’ capacity. However, we should consider that physical exercise might affect cognitive functions only in the ages in which these functions are subjected to development or are in decline (age dependence hypothesis; [25]). Best [60] suggested that an immature neuronal circuit is susceptible to experiences and may be more easily affected by physical exercise than already mature brain structures. Hötting and Röder [25] suggested that the cognitive functions undergoing developmental changes (e.g., executive functions in childhood and old age) could receive more significant benefits from exercise.

## 6. Conclusions

This study investigated the effect of one bout of open- or closed-skill exercise on STM subsystems in primary school-aged children in the school context. A beneficial effect of open-skill exercise in children aged 7–10 years and of closed-skill exercise for older children (9–10 years) on STM retention emerged compared to the control activity. Results contribute to the mounting evidence for the selective nature of the exercise–cognition link and present novel findings on the benefits of valid ecological settings at school. We concentrated on ordinary open- and closed-skill exercises already widely used in schools [61], demonstrating their valuable impact on WM’s subsystems. This study extended our findings of the benefits of one bout of different types of physical activity (i.e., open- and closed-skill exercise [42]) to primary school children. These results are interesting as late childhood is a sensitive period of cognitive development. Thus, these findings might be central for the organisation of cost-effective learning-supportive programs aimed at developing specific cognitive domains, as single bouts of exercise improving WM abilities may, in turn, increase academic performance in the long term. Indeed, WM underlies everyday functioning and academic achievement [54], and linearly develops from preschool through adolescence [60]. Our results might further support research showing that open-skill, single bouts of exercise at school might be promising interventions to promote children’s cognitive development [62] as it could easily be implemented into children’s daily school routines [61,63,64]. In particular, an interesting aspect of this study is related to the use of an intermediate duration of exercise (i.e., 30 min) compared to the high duration used in other studies [10,65,66]. This duration turned out to be effective in children and may suggest that half an hour of targeted physical activity can be promising in terms of applicability to various school settings.

However, some limitations must be considered. This study limited data collection to only a few classes (3rd and 4th), and future directions might also include older children and pre-adolescents to have a more precise developmental trajectory of the effect of physical activity on WM as children of different ages might be differentially sensitive according to the age dependence hypothesis [25]. Moreover, we did not compare other WM measures to investigate the relationships among the STM subsystems and the central executive capacities, and future studies might address this issue. Lastly, we did not insert a third measurement time (for example, 30 min or one hour after the exercise bout) to investigate how long the positive effect on cognitive functioning lasts.

To conclude, aerobic activity should become a fundamental part of school-age children’s daily lives. These findings should encourage parents and educators to reconsider the importance of aerobic exercise and regular physical activity not only for physical development but also for cognitive development.

## Data Availability

The data presented in this study are available on request from the corresponding author.

## References

[1] Biddle S.J., Fox K., Boutcher S. (2003). Physical Activity and Psychological Well-Being.

[2] Ealves H., Voss M.W., Boot W.R., Edeslandes A., Ecossich V., Salles J.E., Kramer A.F. (2013). Perceptual-Cognitive Expertise in Elite Volleyball Players. Front. Psychol..

[3] Budde H., Voelcker-Rehage C., Pietraßyk-Kendziorra S., Ribeiro P., Tidow G. (2008). Acute coordinative exercise improves attentional performance in adolescents. Neurosci. Lett..

[4] Ottoboni G., Nicoletti R., Tessari A. (2021). The Effect of Sport Practice on Enhanced Cognitive Processing of Bod-ily Indices: A Study on Volleyball Players and Their Ability to Predict Hand Gestures. Int. J. Environ. Res. Public Health.

[5] Tessari A., Lugli L., Nicoletti R., Ricciardelli P. (2021). Do boxing athletes differ from controls in visually analysing opponent’s postures? A pilot study tracking eye movements. J. Hum. Sport Exerc..

[6] Hillman C.H., Erickson K.I., Kramer A. (2008). Be smart, exercise your heart: Exercise effects on brain and cognition. Nat. Rev. Neur Sci..

[7] Sibley B.A., Beilock S.L. (2007). Exercise and working memory: An individual differences investigation. J. Sport Exerc. Psychol..

[8] Tomporowski P.D. (2003). Effects of acute bouts of exercise on cognition. Acta Psychol..

[9] Bidzan-Bluma I., Lipowska M. (2018). Physical activity and cognitive functioning of children: A systematic review. Int. J. Environ. Res. Public Health.

[10] Pesce C., Crova C., Cereatti L., Casella R., Bellucci M. (2009). Physical activity and mental performance in preadolescents: Effects of acute exercise on free-recall memory. Ment. Health Phys. Act..

[11] Ellemberg D., St-Louis-Deschênes M. (2010). The effect of acute physical exercise on cognitive function during development. Psychol. Sport Exerc..

[12] Davis C.L., Tomporowski P.D., Boyle C.A., Waller J.L., Miller P.H., Naglieri J.A., Gregoski M. (2007). Effects of Aerobic Exercise on Overweight Children’s Cognitive Functioning: A Randomized Controlled Trial. Res. Q. Exerc. Sport.

[13] Hinkle J.S., Tuckman B.W., Sampson J.P. (1993). The Psychology, Physiology, and Creativity of Middle School Aerobic Exercisers. Elem. Sch. Guid. Couns..

[14] Lambrick D., Stoner L., Grigg R., Faulkner J. (2016). Effects of continuous and intermittent exercise on executive function in children aged 8–10 years. Psychophysiol..

[15] Audiffren M. (2009). Acute Exercise and Psychological Functions: A Cognitive-Energetic Approach. Exercise and Cognitive Function.

[16] McMorris T., Sproule J., Turner A., Hale B.J. (2011). Acute, intermediate intensity exercise, and speed and accuracy in working memory tasks: A meta-analytical comparison of effects. Physiol. Behav..

[17] Coles K., Tomporowski P.D. (2008). Effects of acute exercise on executive processing, short-term and long-term memory. J. Sports Sci..

[18] Audiffren M., Tomporowski P.D., Zagrodnik J. (2008). Acute Aerobic exercise and information processing: Energising motor processes during a choice reaction time task. Acta Psychol..

[19] Singh A., Uijtdewilligen L., Twisk J.W., Van Mechelen W., Chinapaw M.J. (2012). Physical activity and performance at school: A systematic review of the literature including a methodological quality assessment. Arch. Pediatr. Adolesc. Med..

[20] Barenberg J., Berse T., Dutke S. (2011). Executive functions in learning processes: Do they benefit from physical activity?. Educ. Res. Rev..

[21] Best J.R. (2010). Effects of physical activity on children’s executive function: Contributions of experimental research on aerobic exercise. Dev. Rev..

[22] Roberts G., Quach J., Spencer-Smith M., Anderson P.J., Gathercole S., Gold L., Sia K.-L., Mensah F., Rickards F., Ainley J. (2016). Academic outcomes 2 years after working memory training for children with low working memory: A randomised clinical trial. JAMA Pediatr..

[23] Chen K., Chan A.H.S. (2014). Gerontechnology acceptance by elderly Hong Kong Chinese: A senior technology acceptance model (STAM). Ergonomics.

[24] Kamijo K., Pontifex M.B., O’Leary K.C., Scudder M.R., Wu C.-T., Castelli D.M., Hillman C.H. (2011). The effects of an afterschool physical activity program on working memory in pre-adolescent children. Dev. Sci..

[25] Hötting K., Röder B. (2013). Beneficial effects of physical exercise on neuroplasticity and cognition. Neurosci. Biobehav. Rev..

[26] Aronen E., Vuontela V., Steenari M.-R., Salmi J., Carlson S. (2005). Working memory, psychiatric symptoms, and academic performance at school. Neurobiol. Learn. Mem..

[27] Passolunghi M.C., Siegel L.S. (2001). Short-Term Memory, Working Memory, and Inhibitory Control in Children with Difficulties in Arithmetic Problem Solving. J. Exp. Child Psychol..

[28] Baddeley A. (2010). Working Memory. Curr. Biol..

[29] Cubelli R., Marchetti C., Boscolo G., Della Sala S. (2000). Cognition in Action: Testing a Model of Limb Apraxia. Brain Cogn..

[30] Rumiati R.I., Tessari A. (2002). Imitation of novel and well-known actions. Exp. Brain Res..

[31] Tessari A., Rumiati R.I. (2002). Motor distal component and pragmatic representation of objects. Cog. Brain Res..

[32] Tessari A., Rumiati R.I. (2004). The Strategic Control of Multiple Routes in Imitation of Actions. J. Exp. Psychol. Hum. Percept. Perform..

[33] Gu Q., Zou L., Loprinzi P.D., Quan M., Huang T. (2019). Effects of open versus closed skill exercise on cognitive function: A systematic review. Front. Psychol..

[34] Tenenbaum G. (2003). Expert athlete: An integrated approach to decision making. Expert Performance in Sports: Advances in Research on Sport Expertise.

[35] Williams A.M., Davids K., Williams J.G.P. (1999). Visual Perception and Action in Sport.

[36] Huang C.-J., Lin P.-C., Hung C.-L., Chang Y.-K., Hung T.-M. (2014). Type of physical exercise and inhibitory function in older adults: An event-related potential study. Psychol. Sport Exerc..

[37] Piepmeier A.T., Etnier J.L. (2015). Brain-Derived Neurotrophic Factor (BDNF) as a potential mechanism of the effects of acute exercise on cognitive performance. J. Sport Health Sci..

[38] McAllister A.K., Katz L.C., Lo D.C. (1999). Neurotrophins and synaptic plasticity. Annu. Rev. Neurosci..

[39] Cao L., Liu X., Lin E.-J.D., Wang C., Choi E.Y., Riban V., Lin B., During M.J. (2010). Environmental and genetic activation of a brain-adipocyte bdnf/leptin axis causes cancer remission and inhibition. Cell.

[40] Davis C.L., Lambourne K., McMorris T., Tomporowski P.D., Audiffren M. (2009). Exercise and cognition in children. Exercise and Cognitive Function.

[41] Hung L., Au-Yeung A., Helmer C., Ip A., Elijah L., Wilkins-Ho M., Chaudhury H. (2018). Feasibility and acceptability of an iPad intervention to support dementia care in the hospital setting. Contemp. Nurse.

[42] O’Brien J., Ottoboni G., Tessari A., Setti A. (2017). One bout of open skill exercise improves cross-modal perception and immediate memory in healthy older adults who habitually exercise. PLoS ONE.

[43] Halford G.S., Goswami U. (2002). Information-processing models of cognitive development. Blackwell Handbook of Childhood Cognitive Development.

[44] Wechsler D. (2003). Wechsler Intelligence Scale for Children–Fourth Edition: Technical and Interpretive Manual.

[45] Milner B. (1971). Interhemispheric differences in the localisation of psychological processes in man. Br. Med. Bull..

[46] Corsi P.M. (1973). Human memory and the medial temporal region of the brain. Diss. Abstr. Int..

[47] Tessari A., Toraldo A., Lunardelli A., Zadini A., Rumiati R.I. (2015). STIMA: A short screening test for ideo-motor apraxia, selective for action meaning and bodily district. Neurol. Sci..

[48] Kephart N.C. (1971). The Slow Learner in the Classroom.

[49] Schofield W.N. (1976). Do Children Find Movements Which Cross the Body Midline Difficult?. Q. J. Exp. Psychol..

[50] Brass M., Bekkering H., Wohlschläger A., Prinz W. (2000). Compatibility between Observed and Executed Finger Movements: Comparing Symbolic, Spatial, and Imitative Cues. Brain Cogn..

[51] Van der Niet A.G., Smith J., Scherder E.J., Oosterlaan J., Hartman E., Visscher C. (2015). Associations between daily physical activity and executive functioning in primary school-aged children. J. Sci. Med. Sport.

[52] Wang M.V., Lekhal R., Aaro L.E., Holte A., Schjolberg S. (2014). The Developmental relationship between language and motor performance from 3 to 5 years of age: A prospective longitudinal population study. BMC Psychol..

[53] Roberts K.L., Strait J.A.E., Decker S.L. (2018). Developmental trajectories of verbal, static visual-spatial, and dynamic visual-spatial working memory. Contemp. Sch. Psychol..

[54] Gathercole S., Pickering S.J., Ambridge B., Wearing H. (2004). The Structure of working memory from 4 to 15 years of age. Dev. Psychol..

[55] O’Brien J., Ottoboni G., Tessari A., Setti A. (2021). Multisensory Perception, Verbal, Visuo-spatial and motor working memory modulation after a single open- or closed-skill exercise session in children. J. Cogn. Enhanc..

[56] Kramer A.F., Colcombe S. (2018). Fitness effects on the cognitive function of older adults: A meta-analytic study—Revisited. Perspect. Psychol. Sci..

[57] Tomporowski P.D., Ellis N.R., Stephens R. (1987). The immediate effects of strenuous exercise on free-recall memory. Ergonomics.

[58] Tomporowski P.D., Ganio M.S. (2006). Short? Term effects of aerobic exercise on executive processing, memory, and emotional reactivity. Int. J. Sport Exerc. Psychol..

[59] Winter B., Breitenstein C., Mooren F.C., Voelker K., Fobker M., Lechtermann A., Krueger K., Fromme A., Korsukewitz C., Floel A. (2007). High Impact Running Improves Learning. Neurobiol. Learn. Mem..

[60] Best J.R., Miller P.H. (2010). A Developmental Perspective on Executive Function. Child. Dev..

[61] Masini A., Marini S., Gori D., Leoni E., Rochira A., Dallolio L. (2020). Evaluation of school-based interventions of active breaks in primary schools: A systematic review and meta-analysis. J. Sci. Med. Sport.

[62] Diamond A., Lee K. (2011). Interventions Shown to Aid Executive Function Development in Children 4 to 12 Years Old. Science.

[63] Masini A., Lanari M., Marini S., Tessari A., Toselli S., Stagni R., Bisi M.C., Bragonzoni L., Gori D., Sansavini A. (2020). A Multiple Targeted Research Protocol for a Quasi-Experimental Trial in Primary School Children Based on an Active Break Intervention: The Imola Active Breaks (I-MOVE) Study. Int. J. Environ. Res. Public Health.

[64] Masini A., Marini S., Leoni E., Lorusso G., Toselli S., Tessari A., Ceciliani A., Dallolio L. (2020). Active Breaks: A Pilot and Feasibility Study to Evaluate the Effectiveness of Physical Activity Levels in a School Based Intervention in an Italian Primary School. Int. J. Environ. Res. Public Health.

[65] Davis C.L., Tomporowski P.D., McDowell J.E., Austin B.P., Miller P.H., Yanasak N.E., Allison J.D., Naglieri J.A. (2011). Exercise Improves Executive Function and Achievement and Alters Brain Activation in Overweight Children: A Randomised, Controlled Trial. Health Psychol..

[66] Tomporowski P.D., McCullick B., Pendleton D.M., Pesce C. (2015). Exercise and children’s cognition: The role of exercise characteristics and a place for metacognition. J. Sport Health Sci..

